# IL7-Fc Enhances the Efficacy of Adoptive T Cell Therapy under Lymphopenic Conditions in a Murine Melanoma Model

**DOI:** 10.3390/cells10082018

**Published:** 2021-08-07

**Authors:** Eun M. Yu, Eunjung Cho, Rohit Singh, Seon-Hee Kim, Chungyong Han, Seongeun Han, Don G. Lee, Young H. Kim, Byoung S. Kwon, Beom K. Choi

**Affiliations:** 1 Biomedicine Production Branch, Program for Immunotherapy Research, National Cancer Center, Goyang 10408, Korea; emy@ncc.re.kr (E.M.Y.); dgilee@ncc.re.kr (D.G.L.); yhokim@eutilex.com (Y.H.K.); 2 Division of Tumor Immunology, National Cancer Center, Goyang 10408, Korea; 75086@ncc.re.kr (E.C.); rohit@ncc.re.kr (R.S.); 73535@ncc.re.kr (S.-H.K.); chungyong.han27@gmail.com (C.H.); 74630@ncc.re.kr (S.H.); 3 Eutilex, Co., Ltd., Geumcheon-gu, Seoul 08594, Korea; bskwon@eutilex.com; 4 Department of Medicine, Tulane University Health Sciences Center, New Orleans, LA 70112, USA

**Keywords:** IL-7, recombinant IL7-Fc, IL-2, immunotherapy, melanoma, adoptive cell therapy, lymphopenia, tumor-specific T cells

## Abstract

Adoptive cell therapy (ACT) using tumor-reactive T cells is a promising form of immunotherapy to specifically target cancer. However, the survival and functional maintenance of adoptively transferred T cells remains a challenge, ultimately limiting their efficacy. Here, we evaluated the use of recombinant IL7-Fc in ACT. In a lymphopenic murine melanoma model, IL7-Fc treatment led to the enhanced inhibition of tumor growth with an increased number of adoptively transferred CD8^+^ T cells in tumor tissue and tumor-draining lymph nodes. Additionally, IL7-Fc further enhanced anti-tumor responses that were induced by recombinant human IL2 in the same mouse model. In contrast, in an immunocompetent murine melanoma model, IL7-Fc dampened the anti-tumor immunity. Further, IL7-Fc decreased the proliferation of adoptively transferred and immune-activated tumor-reactive CD8^+^ T cells in immunocompetent mice by inducing the massive expansion of endogenous T cells, thereby limiting the space for adoptively transferred T cells. Our data suggest that IL7-Fc is principally beneficial for enhancing the efficacy of tumor-reactive T-cells in lymphopenic conditions for the ACT.

## 1. Introduction

Adoptive cell therapy (ACT) using T cells is a promising treatment modality for cancer that involves the administration of tumor-reactive T cells that attack malignant cells [1]. However, in cancer patients, immune-suppressive elements, including regulatory T cells (Tregs) and myeloid-derived suppressor cells (MDSCs), and an increase in inhibitory molecules limit adequate T cell function [2]. Moreover, the heterogeneity of cancer clones and immune evasion of tumor cells are other hurdles that limit the efficacy of ACT by inducing cancer recurrence in patients [3]. To resolve these issues, transferred T cells should continuously produce effector T cells by successfully converting some of these cells into memory T cells and self-sustaining the cancer immunity cycle [4].

The standard ACT regimen includes prior-lymphodepletion with cytotoxic agents, including cyclophosphamide (CTX) and fludarabine, and subsequent administration of recombinant human IL-2 (rhIL-2) after T cell transfer [5]. IL-2 is predominantly secreted by activated CD4^+^ T cells and stimulates the growth of activated CD8^+^ T cells expressing the IL-2 receptors containing α, β, and γ chains [6]. However, IL-2 is also crucial for the maintenance of CD4^+^ Treg cells [7], and its half-life in humans is approximately 7 min [8]. Consequently, IL-2 should be repeatedly administered to patients receiving ACT at a higher dose, which frequently causes multiple adverse effects, including fever, chills, malaise, arthralgias, and unexpected capillary leak, and is suspected to induce the expansion of Treg cells [8].

Although the clinical benefit of ACT is improved by utilizing IL-2, a more reliable and safer modality to improve the efficacy of ACT is still required. To improve the efficacy of ACT, transferred T cells should successfully generate memory T cells [9,10]. Prior lymphodepletion removes homeostatic cytokine sinks, thereby improving the efficacy of ACT using tumor-specific CD8^+^ T cells [11]. Homeostatic cytokines, such as IL-7 and IL-15, are involved in improving the efficacy of ACT. Although it is reported that lymphodepleting chemotherapy does not lead to increased availability of the endogenous IL-7 in mice [12], enhancing IL-7-mediate signaling appears to be beneficial in augmenting the efficacy of ACT. IL-15 is known to promote the generation of long-lived memory T cells with superior functional capacity and potential use in adoptive T-cell transfer protocols [13].

IL-7 is a hematopoietic cytokine involved in regulating multiple aspects of T cell biology, including survival, homeostasis, metabolism, and memory [14,15]. A limited amount of IL-7 is produced by non-hematopoietic cells and is consumed by various types of cells, including T cells [16]. Although IL-7 is essential for T cell survival as well as homeostatic proliferation [14], IL-7R expression is restricted in naïve and memory T cells; therefore, we questioned whether the efficacy of ACT using activated T cells would be improved with IL-7 utilization, particularly with the Fc fusion protein of IL-7 (IL7-Fc), which has an increased half-life and signaling strength.

Previous studies indicate that systemic treatment of IL7-Fc induces proliferation of CD8^+^ T cells [17] and improves anti-tumor responses by increasing CD8^+^ T cells in the periphery and by promoting the accumulation of CD8^+^ T cells in tumor tissues [18]. Moreover, Kim et al. showed that IL7-Fc was much effective in profoundly suppressing tumor growth in combination with cyclophosphamide [18]. Consistent with these studies, IL7-Fc also significantly increases the number of CD8^+^ and CD4^+^ T cells in healthy adult human volunteers [19].

Here, we provide evidence that IL7-Fc successfully enhances the proliferation of both naïve and activated CD8^+^ T cells under lymphopenic conditions. However, under non-lymphopenic conditions, IL7-Fc promotes the homeostatic proliferation of naïve CD8^+^ T cells, but restricts the proliferation of activated CD8^+^ T cells and eventually promotes tumor growth. Our data suggest that IL7-Fc would be clinically beneficial in cancer patients receiving adoptively transferred T cells under lymphopenic conditions.

## 2. Materials and Methods

### 2.1. Reagents

Mouse gp100_25–33_ (mgp100, EGSRNQDWL) and human gp100_25–33_ (hgp100, KVPRNQDWL) peptides were synthesized by Peptron (Daejeon, Korea). CD8 microbeads were purchased from Miltenyi Biotec (Auburn, AL, USA). All antibodies used for flow cytometry were purchased from BD Bioscience, including anti-Thy1.1-FITC, anti-CD3-FITC, anti-B220-FITC, anti-CD62L-FITC, anti-Ly-6C-FITC, anti-CD19-PE, anti-CD8-PE, anti-CD44-PE, anti-CD8-PE-Cy5, anti-Thy1.1-PE-Cy5, anti-CD11b-PE-Cy5, anti-CD4-APC, anti-CD8-APC, and anti-CD45-APC. Recombinant human IL-2 (Proleukin) was purchased from Novartis, and the Fc fusion protein of human IL-7 (IL7-Fc), which consist of the extracellular domain of human IL-7 (aa 26–177) fused to the N-terminus of the Fc portion of a mutant human IgG1, was obtained from AdipoGen (Seoul, Korea). The CellTrace CFSE Cell Proliferation Kit was purchased from Invitrogen (Carlsbad, CA, USA).

### 2.2. Mice

Six to eight-week-old C57BL/6 mice were purchased from OrientBio (Gapyeong, Korea) while B6.Cg-Thy1^a^/Cy Tg(TcraTcrb)8Rest/J (pmel-1 Thy1.1^+^) transgenic (Tg) and B6.129S7-Rag1^tm1Mom^/J (RAG1^−/−^) knockout (KO) mice were procured from the Jackson Laboratory (JAX; Bar Harbor, ME, USA). All mice were maintained under specific-pathogen-free conditions at the animal facility of the National Cancer Center in Korea. The procedures were approved by the Institutional Animal Care and Use Committee (IACUC) of the National Cancer Center Institute (NCC-21-632). Animal experiments were conducted according to the Guidelines on the Care and Use of Laboratory Animals of the Institute of Laboratory Animal Resources (ILAR).

### 2.3. Adoptive T Cell Therapy Model and IL7-Fc Treatment

B16-F10 melanoma cells were injected subcutaneously (s.c.) into the backs of C57BL/6 mice (2 × 10^5^ cells per mouse). Three days later, mice were injected intraperitoneally (i.p.) with 0, 150, or 300 mg/kg of CTX to induce lymphopenia. To prepare activated pmel-1 Thy1.1^+^CD8^+^ T cells, CD8^+^ T cells were isolated from the lymph nodes (LNs) and spleen of pmel-1 Thy1.1^+^ Tg mice using mouse CD8-microbeads (Miltenyi Biotec, Auburn, CA, USA) according to the manufacturer’s instructions, and activated in high glucose DMEM supplemented with 10% FBS and antibiotics in the presence of 5 μg of human gp100 (hgp100) peptide and 5% of CD8-depleted splenocytes for 2 days. Activated pmel-1 Thy1.1^+^CD8^+^ T cells were injected into mice via the intravenous (i.v.) route (2 × 10^6^ cells per mouse) on day 5, and 25,000 IU of recombinant human IL-2 (rhIL-2) and/or 1 μg of IL7-Fc were administered daily to mice for 6 days.

### 2.4. Flow Cytometry

Single cell suspensions were prepared from inguinal tumor-draining lymph nodes (TDLNs) and spleen, and the cells were pre-incubated with an Fc blocker (2.4G2 clone) for 5 min. The cells were further stained with anti-Thy1.1-FITC and anti-CD8-PE-Cy5 to discriminate the transferred pmel-1 CD8^+^ T cells or with anti-CD44-PE, anti-CD62L-FITC, and anti-Thy1.1-PE-Cy5 along with anti-CD4-APC or anti-CD8-APC to confirm the activation status of endogenous T cells. For monocytes and B cells in the spleen and bone marrow (BM), single-cell suspensions of spleen and BM were pre-incubated with Fc blocker for 5 min and further stained with anti-CD11b-PE-Cy5 and anti-B220-FITC.

### 2.5. Calculation of the Absolute Cell Numbers

The absolute cell numbers of TDLN, spleen, and BM were calculated using an ADAM-MC2 automatic cell counter (NanoEntek, Seoul, Korea) according to the manufacturer’s instructions. Absolute cell numbers of each population were calculated as follows: absolute cell number = % of measured cells × total cells/100).

### 2.6. Monitoring the Repopulation of Leukocytes in Peripheral Blood

C57BL/6 mice were injected i.p. with 300 mpk CTX to induce transient leukopenia 10 days after the B16-F10 melanoma challenge. CTX-treated mice were i.p. injected with 1 μg of IL7-Fc three times from day 12 or injected once with 25 μg of IL7-Fc on day 12. Heparinized blood samples were routinely collected via retro-orbital bleeding at 0, 2, 4, 7, 9, and 11 days after CTX treatment and directly stained with anti-Ly-6C-FITC, anti-CD11b-PE, anti-CD3-FITC, anti-CD19-PE, and anti-CD45-APC. All samples were subsequently analyzed using FACSCalibur (BD Bioscience).

### 2.7. CFSE Dilution Assay of pmel-1 CD8^+^ T Cells in WT and RAG 1^−/−^ Mice

For the homeostatic proliferation of naïve CD8^+^ T cells, CD8^+^ T cells were isolated from the LNs and spleen of pmel-1 Thy1.1^+^ Tg mice using mouse CD8-microbead (Miltenyi Biotec, Auburn, CA, USA) and labeled with CellTrace CFSE kit (Invitrogen, Eugene, OR, USA). WT or RAG1^−/−^ C57BL/6 mice were injected i.v. with 5 × 10^5^ or 2 × 10^6^ CFSE-labeled pmel-1 Thy1.1^+^CD8^+^ T cells, respectively, and daily injected i.p. with 1 μg of hIgG or IL7-Fc for 3 days. Inguinal TDLNs were collected from mice on day 7, and single-cell suspensions were stained with anti-Thy1.1-PE-Cy5 and anti-CD8-PE.

For Ag-dependent proliferation of CD8^+^ T cells, WT or RAG1^−/−^ C57BL/6 mice were injected i.v. with 5 × 10^5^ or 2 × 10^6^ CFSE-labeled pmel-1 Thy1.1^+^CD8^+^ T cells, respectively, immunized s.c. with 20 μg of mgp100 peptide emulsified in incomplete Freund’s adjuvant, and i.p. administered 1 μg of hIgG or IL7-Fc daily for 3 days. Inguinal TDLNs were collected from mice on day 4, and single cell suspensions were stained with anti-Thy1.1-PE and anti-CD8-PE-Cy5. All samples were subsequently analyzed using FACSCalibur (BD Bioscience).

## 3. Results

### 3.1. The Fc Fusion Protein of IL-7 Enhances the Anti-Tumor Effect of Activated pmel-1 CD8^+^ T Cells Only with Prior Lymphodepletion

**Figure 1:**
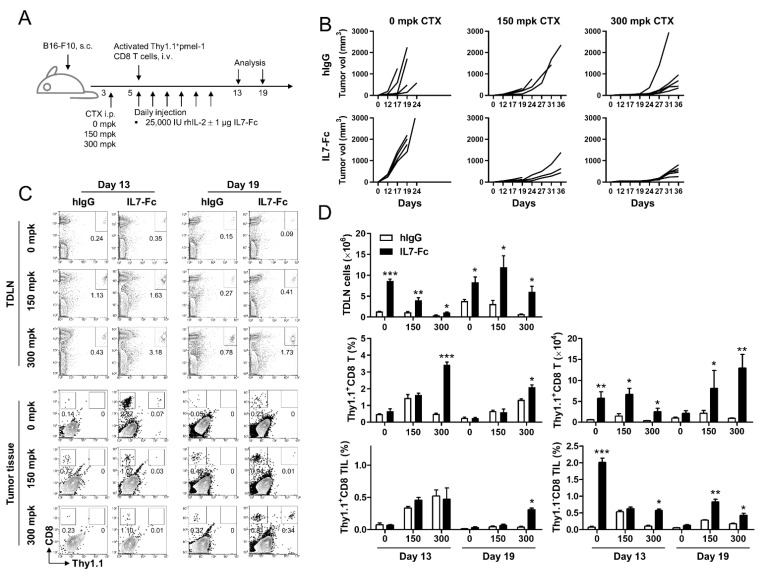
Anti-tumor effects of IL7-Fc in non-lymphopenic and lymphopenic conditions. (**A**) C57BL/6 mice were subcutaneously implanted with 2 × 10^5^ B16-F10 melanoma cells in their backs. Mice were i.p. injected with the indicated dose of CTX to induce prior lymphodepletion on day 3. Activated pmel-1 Thy1.1^+^CD8^+^ T cells were prepared by isolating CD8^+^ T cells from Thy1.1^+^pmel-1 Tg mice and stimulating them with 5 μg/mL of human gp100 peptide in the presence of 5% CD8-depleted splenocytes for 2 days, and transferred into mice on day 5 via the i.v. route. All mice received 25,000 IU of rhIL-2 daily for 6 days. Human IgG (hIgG) or IL7-Fc was also injected daily into mice for 6 days. (**B**) The growth rate of B16-F10 melanoma. (**C**) Flow cytometric analysis of tumor-draining lymph nodes (TDLNs) and tumor tissues. Single cell suspensions were prepared from inguinal TDLNs and tumor tissues on days 13 and 19, and stained with anti-CD8-PE-Cy5 and anti-Thy1.1-FITC. All samples were further stained with propidium iodide (PI) and subsequently analyzed using FACSCalbur (BD Bioscience). Live cells were gated and plotted as the indicated markers. (**D**) Absolute numbers of TDLN cells were calculated using ADAM-MC2 (Nanoentek, Seoul, Korea). Absolute numbers of Thy1.1^+^CD8^+^ T cells were calculated from TDLN cell numbers and the percentages of Thy1.1^+^CD8^+^ T cells. Percentages of transferred pmel-1 Thy1.1^+^CD8^+^ T cells and endogenous Thy1.1^−^CD8^+^ T cells in tumor tissues were calculated from (**C**). CTX; cyclophosphamide, mpk; mg per Kg. Data are from three (**B**) or two (**C**,**D**) independent experiments with 5 (**B**) or 3 (**C**,**D**) mice per experiment. Student’s *t*-test was performed in D, and the results are expressed as mean ± SD (* *p* < 0.05; ** *p* < 0.01; *** *p* < 0.005).

**Figure 2:**
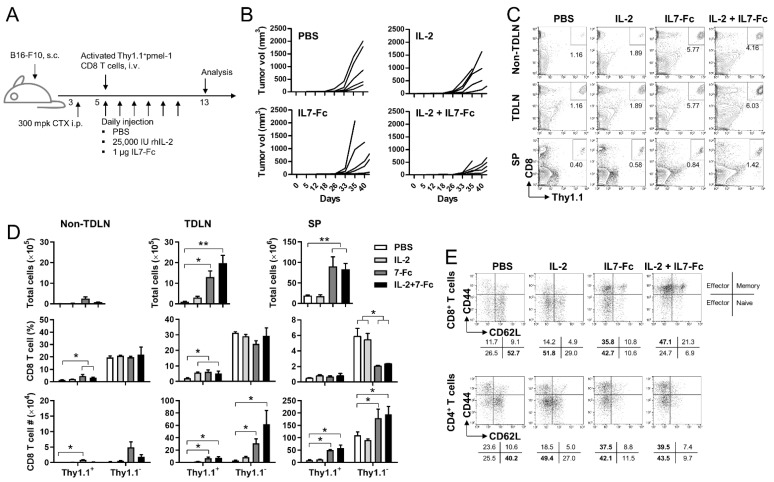
Anti-tumor effects of IL7-Fc with IL-2 in lymphopenia-induced B16-F10 melanoma-bearing B6 mice. (**A**) B16-F10 melanoma-bearing C57BL/6 mice were i.p. injected with 300 mpk of CTX on day 3 followed by 2 × 10^6^ activated pmel-1 Thy1.1^+^CD8^+^ T cells, and further treated with PBS, rhIL-2, hIL7-Fc, or rhIL-2 plus hIL7-Fc as described above. (**B**) The growth rate of B16-F10 melanoma cells. (**C**) Inguinal TDLN, popliteal non-TDLN, and spleen (SP) were collected from each group of mice on day 13 and single cell suspensions were stained with anti-CD8-PE-Cy5 and anti-Thy1.1-FITC. All samples were further stained with propidium iodide (PI) and subsequently analyzed using FACSCalbur (BD Bioscience). Live cells were gated and plotted as the indicated markers. (**D**) Absolute numbers of live cells in non-DLN, TDLN, and spleen. Percentages and absolute numbers of CD8^+^ T cells, the transferred pmel-1 Thy1.1^+^CD8^+^ T cells, and the endogenous Thy1.1^−^CD8^+^ T cells in non-TDLN, TDLN, and spleen on day 13. (**E**) TDLN cells were stained with anti-CD44-PE, anti-CD62L-FITC, and anti-Thy1.1-PE-Cy5 along with anti-CD4-APC or anti-CD8-APC. Gated Thy1.1-negative CD4^+^ or CD8^+^ cells were plotted as CD44 vs. CD62L. Data are from three independent experiments with 5 (**B**) or 3 (**C**,**D**) mice per experiment. Student’s *t*-test was performed in D, and the results are expressed as mean ± SD (* *p* < 0.05; ** *p* < 0.01).

As ACT using T cells essentially requires prior lymphodepletion [5], we first examined the anti-tumor effects of the Fc fusion protein of IL-7 (IL7-Fc) under none, mild, and severe lymphopenic conditions. As the IL7-Fc we used for the animal experiment has human IgG1 and mutations in the Fc part in the complement (C1q) and FcgR I binding sites of the IgGs, this IL7-Fc is expected to minimally mediate antibody directed cytotoxicity (ADCC) and complement directed cytotoxicity (CDC) and thus, exclusively prolongs signaling through IL-7R. B16-F10 melanoma-bearing C57BL/6 mice were treated with 0, 150, or 300 mg per kg (mpk) of CTX on day 3 and further injected with activated pmel-1 Thy1.1^+^CD8^+^ T cells on day 5. Recombinant human IL-2 was administered daily with or without recombinant IL7-Fc for 6 days from day 5 (Figure 1A). Unexpectedly, the administration of IL7-Fc exacerbated the growth of B16-F10 melanoma in immune-competent B6 mice (Figure 2B; left panel) but synergistically suppressed tumor growth in mice administered 150 or 300 mkp CTX (Figure 2B; middle and right panels).

Flow cytometry revealed that IL7-Fc increased the percentage of transferred activated pmel-1 Thy1.1^+^CD8^+^ T cells compared with that in hIgG-treated mice under lymphopenic conditions on days 13 and 19 in inguinal TDLNs (Figure 1C). Analysis of tumor-infiltrating lymphocytes (TILs) indicated that IL7-Fc injection did not increase the transfer of Thy1.1^+^CD8^+^ T cells, but increased the infiltration of endogenous Thy1.1^−^CD8^+^ T cells in immune-competent mice on day 13; this increase was impaired by inducing lymphopenia (Figure 1C). However, on day 19, IL7-Fc injection not only enhanced the infiltration of endogenous Thy1.1^−^CD8^+^ T cells but also increased the number of transferred Thy1.1^+^CD8^+^ T cells with severe prior lymphodepletion (Figure 1C).

Statistical analysis indicated that the administration of IL7-Fc significantly increased the total number of TDLN cells in immune-competent mice on day 13; however, this effect was dose-dependently impaired by CTX treatment (Figure 1D). On day 19, total TDLN cell numbers were increased in 0 or 150 mpk CTX-treated mice compared with that of day 13, but minimally in 300 mpk CTX-treated mice (Figure 1D). The administration of IL7-Fc not only accelerated the increase of total TDLN cells in 0 or 1150 mpk CTX-treated mice but also significantly increased TDLN cells in 300 mpk CTX-treated mice (Figure 1D). IL7-Fc injection augmented the percentages of transferred Thy1.1^+^CD8^+^ T cells in TDLNs, which was further enhanced by increasing the CTX dose, which was sustained until day 19 (Figure 1D). Calculation of the absolute cell numbers in TDLN also revealed that IL7-Fc significantly increased the number of transferred Thy1.1^+^CD8^+^ T cells under any conditions on days 13 and 19, and the enhanced expansion of activated pmel-1 CD8^+^ T cells by IL7-Fc lasted longer under lymphopenic conditions (Figure 1D). These data suggest that IL7-Fc promoted the expansion of transferred activated pmel-1 CD8^+^ T cells under lymphopenic conditions (Figure 1D).

TIL analysis revealed that the infiltration of activated Thy1.1^+^CD8^+^ T cells was increased in mice administered 150 or 300 mpk CTX on day 13 independent of IL7-Fc injection; however, IL7-Fc was found to prolong the infiltration of activated pmel-1 CD8^+^ T cells under severe lymphopenic conditions (Figure 1D; bottom). Notably, the infiltration of endogenous Thy1.1^-^CD8^+^ T cells markedly increased in non-treated mice on day 13. This infiltration was also found to be increased by IL7-Fc in CTX-treated mice until day 19 (Figure 1D; bottom).

These data suggest that the transferred activated pmel-1 CD8^+^ T cells were more effectively expanded by IL7-Fc under lymphopenic conditions, and endogenous CD8^+^ T cells that may be in a resting state were well expanded by IL7-Fc independent of lymphopenia. IL7-Fc markedly induced the expansion of endogenous and transferred CD8^+^ T cells and the infiltration of endogenous CD8^+^ T cells into the tumor tissue of immune-competent mice, even on day 13 (Figure 1D). Further, the tumor growth rate was found to be accelerated in these mice (Figure 1B). These data suggest that endogenous CD8^+^ T cells expanded by IL7-Fc in immune-competent mice may minimally include tumor-reactive CD8^+^ T cells. However, in the case of 150 mpk CTX-treated mice, IL7-Fc further suppressed tumor growth rate, but percentages of pmel-1 Thy1.1^+^CD8^+^ TILs were comparable in both hIgG- or IL7-Fc treated mice at day 13 and 19, while endogenous Thy1.1^−^CD8^+^ TILs were still significantly increased by IL7-Fc treatment at day 19, which indicate that IL7-Fc-mediated anti-tumor immunity under lymphopenic condition may include the increase of tumor-reactive endogenous CD8^+^ T cells. Collectively, we conclude that IL7-Fc requires a lymphopenic condition to enhance the anti-tumor response in ACT using T cells.

### 3.2. IL7-Fc in Combination with rhIL-2 Enhance the Anti-Tumor Responses of Activated pmel-1 CD8^+^ T Cells under Lymphopenic Condition

CD25 (IL-2Rα) is primarily expressed on activated CD8^+^ T cells [6] while CD127 (IL-7Rα) is mainly expressed in naïve and memory CD8^+^ T cells [14,15]. Therefore, we investigated whether IL7-Fc enhanced the anti-tumor responses of activated pmel-1 CD8^+^ T cells in combination with rhIL-2. B16-F10 melanoma-bearing B6 mice were injected with 300 mpk CTX on day 3 and activated pmel-1 Thy1.1^+^CD8^+^ T cells on day 5. rhIL-2 and/or IL7-Fc were administered daily to mice from day 5 for 6 days (Figure 2A). As expected, rhIL-2 or IL7-Fc alone suppressed tumor growth rates, but which was further suppressed by the combination of rhIL-2 and IL7-Fc (Figure 2B).

When cervical lymph nodes (which were employed as non-tumor draining lymph nodes (non-TDLNs)), inguinal TDLNs, and spleens were analyzed on day 19, the percentages of transferred pmel-1 CD8^+^ T cells were minimally increased by rhIL-2 and were increased by IL7-Fc in non-TDLN, TDLN, and spleen (Figure 2C). The percentage of transferred pmel-1 CD8^+^ T cells was significantly increased by IL7-Fc in non-TDLN and TDLN; however, when the absolute cell numbers were calculated, the expansion of pmel-1 CD8^+^ T cells was found to be more significant in the TDLN and spleen due to a remarkable increase in total cell numbers (Figure 2D; upper). The absolute numbers of endogenous Thy1.1^-^CD8^+^ T cells were also significantly increased by IL7-Fc in TDLN and spleen, and further increased by the addition of rhIL-2 (Figure 2D; bottom).

As IL7-Fc induced a maximal increase in endogenous Thy1.1^−^CD8^+^ T cells in TDLN and spleen, but not in non-TDLN, we investigated whether IL7-Fc induces the activation and differentiation of endogenous CD8^+^ T cells in TDLN. Flow cytometry indicated that naïve Thy1.1^−^CD8^+^ T cells with the CD44^Low^CD62L^High^ phenotype were the major population in TDLNs of PBS-injected mice; however, effector CD8^+^ T cells with CD44^Low^ or CD44^High^ CD62L^High^ phenotype became a major population in IL7-Fc ± rhIL-2-injected mice (Figure 2E). Similar results were also observed in endogenous CD4^+^ T cells (Figure 2E).

IL7-Fc not only increased the transferred pmel-1 CD8^+^ T cells but also maximally induced the expansion of endogenous CD8^+^ T cells with an effector phenotype, mainly in the TDLN and spleen (Figure 2D). The addition of rhIL-2 further enhanced the IL7-Fc-mediated effects on CD8^+^ T cells, primarily in TLDN. Therefore, these data indicate that IL7-Fc further enhanced rhIL-2-mediated anti-tumor responses by expanding transferred activated pmel-1 CD8^+^ T cells and endogenous CD8^+^ T cells along with their activation and differentiation, which might require the presentation of tumor-associated antigens in TDLN.

### 3.3. IL7-Fc Promotes the Repopulation of B Cells

**Figure 3:**
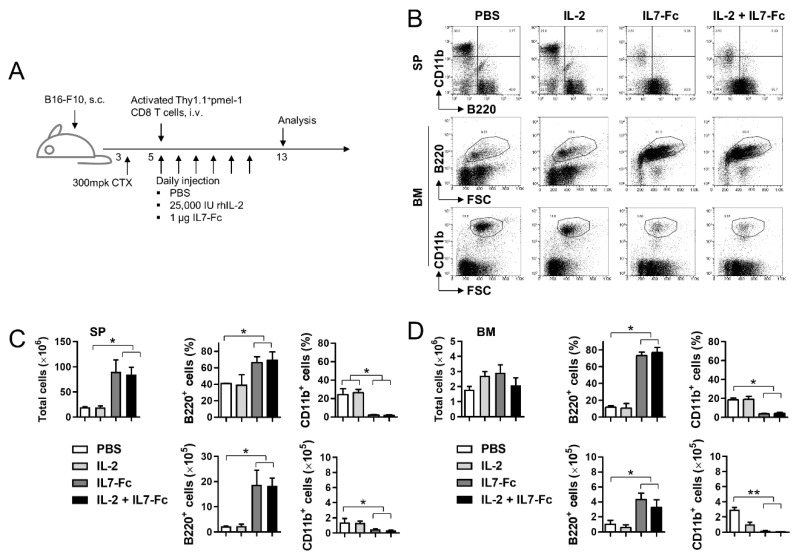
Effects of IL7-Fc on myeloid and lymphoid cells in bone marrow and spleen. (**A**) B16-F10 melanoma-bearing C57BL/6 mice were sequentially injected with CTX, activated pmel-1 Thy1.1^+^CD8^+^ T cells, and further treated with PBS, rhIL-2, hIL7-Fc, or rhIL-2 plus hIL7-Fc as described above. (**B**) BMs and spleens were collected from mice on day 13 and single cell suspensions were stained with anti-CD11b-PE-Cy5 and anti-B220-FITC. All samples were further stained with propidium iodide (PI) and subsequently analyzed using FACSCalbur (BD Bioscience). Live cells were gated and plotted as the indicated markers. (**C**) Absolute numbers of spleen cells were counted, and the percentages and absolute numbers of CD11b^+^ monocytes and B220^+^ B cells were calculated. (**D**) Absolute numbers of BM cells were counted, and the percentages and absolute numbers of CD11b^+^ monocytes and B220^+^ B cells were calculated. Data are from two independent experiments with 3 mice per experiment. Student’s *t*-test was performed in (**C**,**D**), and the results are expressed as mean ± SD (* *p* < 0.05; ** *p* < 0.01).

It is clear that IL7-Fc promotes the expansion of activated and resting CD8^+^ T cells as the number of CD8^+^ T cells was significantly increased by IL7-Fc in TDLN and spleen (Figure 2D; bottom). However, the percentage of CD8^+^ T cells decreased from ~6% to ~2% in the spleen (Figure 2D, middle). This can be explained by an increase in other immune cell subsets. As IL-7 is known to play a crucial role in B cell development by promoting the survival and expansion of lymphoid precursors [20,21,22], we investigated whether IL7-Fc promotes B cell expansion. Tumor-bearing mice received CTX, activated pmel-1 Thy1.1^+^CD8^+^ T cells, and IL-2 and/or IL7-Fc as described above (Figure 3A). On day 13, the percentages of B220^+^ B cells and CD11b^+^ monocytes were ~40% and ~30% in the spleen of PBS-injected mice (Figure 3B). Approximately 40% of B220^+^ B cells reached ~50% by IL-2 and ~60% by IL7-Fc and IL-2 + IL7-Fc (Figure 3B). Conversely, ~30% of the CD11b^+^ monocytes became ~25% by IL-2 and ~3% by IL7-Fc and IL-2 + IL7-Fc (Figure 3B). Similar results (an increase in B220^+^ B cells and a decrease in CD11b^+^ monocytes) were found in the BM (Figure 2B). Statistical analysis indicated that IL7-Fc with or without IL-2 significantly increased total splenocytes and percentages of B220^+^ B cells, and decreased the percentage of CD11b^+^ monocytes in the spleen (Figure 3C). Calculation of absolute cell numbers showed that IL7-Fc induced an approximately 9-fold increase in B cells and a ~3-fold decrease in monocytes in the spleen, independent of IL-2 injection (Figure 3C). Similar results (an increase in B cells and a decrease in monocytes by IL7-Fc with or without IL-2) were also found in BM (Figure 3D).

These data suggest that IL7-Fc promotes the expansion of T/B cells while impeding the development of monocytes.

### 3.4. Repeated Injection of IL7-Fc Causes the Accumulation of Monocytic Cells in Peripheral Blood by Potentially Promoting the Differentiation of Myeloid Cells in Bone Marrow

**Figure 4:**
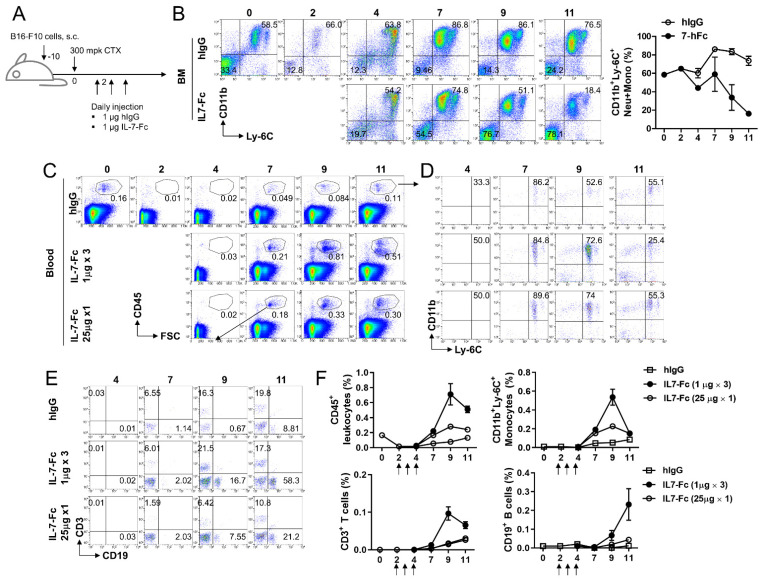
Effects of IL7-Fc on the repopulation of lymphoid and myeloid cells from CTX-mediated lymphopenia. (**A**) B16-F10 melanoma-bearing C57BL/6 mice were injected i.p. with CTX at day 10 and injected daily with hIgG or IL7-Fc from day 12 for 3 days. (**B**) BM cells were collected from mice on the indicated days and stained with anti-CD11b-PE-Cy5 and anti-Ly-6C-FITC. All samples were further stained with propidium iodide (PI) and subsequently analyzed using FACSCalbur (BD Bioscience). Live cells were gated and the frequencies of CD11b^+^Ly-6C^+^ myeloid cells were analyzed. (**C**,**D**) CTX-treated and tumor-bearing mice were i.p. injected with 1 μg of IL7-Fc three times as described above or injected once with 25 μg of IL7-Fc at day 12. Heparinized bloods were collected and directly stained with the indicated antibodies. CD45-gated cells (**C**) were plotted as CD11b vs. CD11b (**D**) or CD3 vs. CD19 (**E**). Percentages of CD45^+^ leukocytes, CD11b^+^Ly-6C^+^ monocytes, CD3^+^ T cells, and CD19^+^ B cells were calculated form (**C**,**E**). Data are from three independent experiments with 3 mice per experiment. Student’s *t*-test was performed in (**B**,**F**), and the results are expressed as mean ± SD.

IL-7 induces myelopoiesis and erythropoiesis; thus, IL-7 administration increases the number of mature myeloid and monocytic cells in the spleen and peripheral blood [23]. Nevertheless, we found a decrease in CD11b^+^ monocytes in the spleen and BM following repeated injection of IL7-Fc (Figure 3C,D). To confirm whether IL7-Fc promotes or diminishes the repopulation of monocytes, CTX was injected into tumor-bearing mice to induce non-myeloablation, and IL7-Fc or human IgG (hIgG) was administered to the mice to induce the repopulation of lymphoid and myeloid cells (Figure 4A). Two days after CTX treatment, flow cytometry confirmed that CD11b^+^Ly-6C^+^ monocytes were successfully depleted in the BM (Figure 4B). CD11b^+^Ly-6C^+^ monocytic cells fully recovered from leukopenia 4 days after CTX treatment and sustained their ratio until day 11 (Figure 4B; upper panel). However, when IL7-Fc was administered to CTX-treated mice, the percentage of CD11b^+^Ly-6C^+^ monocytic cells became normal on day 4, peaked at day 7, and then continuously declined (Figure 4B; lower panel).

We also examined the proportions of leukocytes, lymphocytes, and monocytes in the peripheral blood following CTX treatment. In hIgG-treated mice, the percentage of CD45^+^ leukocytes gradually recovered from CTX-mediated leukopenia and became normal on day 11 (Figure 4C). When tumor-bearing and CTX-treated mice received IL7-Fc under two different conditions: low-dose multiple injections vs. high-dose single injection, the repopulation of CD45^+^ cells was found to be continuously accelerated by both conditions (Figure 4C). However, low-dose multiple injections rather than high-dose single injections were more efficient at enhancing leukocyte repopulation in peripheral blood (Figure 4C). Immune cell subset analysis indicated that IL7-Fc efficiently increased CD11b^+^Ly-6C^+^ monocytic cells, CD3^+^ T, and CD19^+^ B cells (Figure 4D,E). Statistical analysis indicated that the temporal injection of IL7-Fc transiently increased CD45^+^ leukocytes, CD3^+^ T cells, and CD11b^+^Ly-6C^+^ monocytic cells, which peaked at day 9, and continuously increased CD19^+^ B cells (Figure 4F).

These findings suggest that IL7-Fc promotes the repopulation of both lymphoid and myeloid cells from the BM and accumulates them in the periphery. Repeated injection of IL7-Fc decreased CD11b^+^Ly-6C^+^ monocytic cells in BM, which might be due to the enhanced differentiation of monocytic cells from BM.

### 3.5. In the Lymphopenic Environment, IL7-Fc Efficiently Enhances the Proliferation of Activated pmel-1 CD8^+^ T Cells, Which Is Impeded in the Non-Lymphopenic Environment

**Figure 5:**
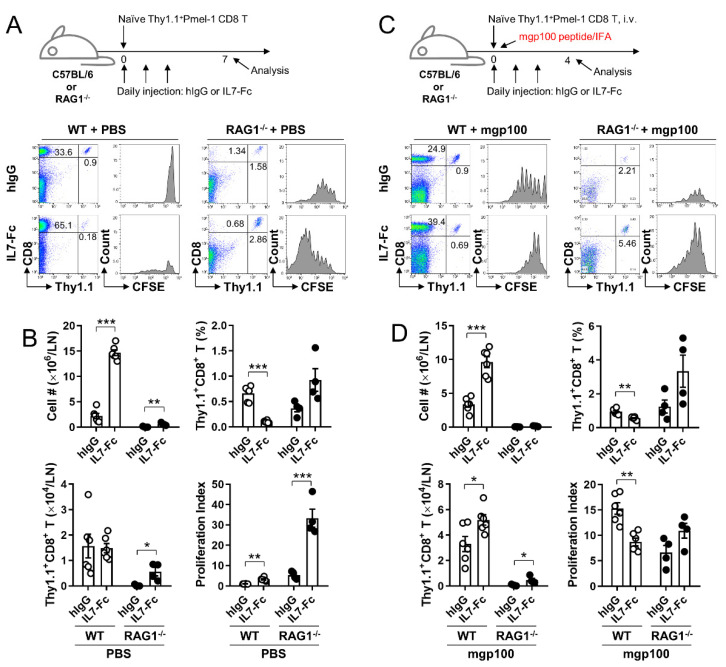
Effects of IL7-Fc on homeostatic and Ag-dependent proliferation of pmel-1 CD8^+^ T cells in non-lymphopenic and lymphopenic conditions. (**A**) WT or RAG1^−/−^ C57BL/6 mice were injected i.v. with 5 × 10^5^ of CFSE-labeled pmel-1 Thy1.1^+^CD8^+^ T cells and daily injected i.p. with 1 μg of hIgG or IL7-Fc for 3 days. After seven days, a single cell suspension of inguinal LN cells was stained with anti-CD8-PE and anti-Thy1.1-PE-Cy5. CFSE dilution rates of the gated Thy1.1^+^CD8^+^ cells were assessed by flow cytometry (**A**). (**B**) Total numbers of live LN cells, percentages of Thy1.1^+^CD8^+^ T cells, and absolute numbers of Thy1.1^+^CD8^+^ T cells were determined by flow cytometry and cell counting. Proliferation index was calculated using Flow Jo software. (**C**,**D**) WT or RAG1^−/−^ C57BL/6 mice were administered 5 × 10^6^ of CFSE-labeled pmel-1 Thy1.1^+^CD8^+^ T cells, immunized s.c. with mgp100 peptide emulsified in IFA, and injected daily with hIgG or IL7-Fc for 3 days. After four days, the CFSE dilution rates of the Thy1.1^+^CD8^+^ cells in inguinal LNs were assessed by flow cytometry (**C**). (**D**) Total numbers of live LN cells, percentages of Thy1.1^+^CD8^+^ T cells, absolute numbers of Thy1.1^+^CD8^+^ T cells, and proliferation index were calculated as described above. Data are from two independent experiments with 3 mice per experiment. Student’s *t*-test was performed in (**B**,**D**), and the results are expressed as mean ± SD (* *p* < 0.05; ** *p* < 0.01; *** *p* < 0.005).

As IL7-Fc more efficiently increased the infiltration of endogenous CD8^+^ T cells in the non-lymphopenic rather than the lymphopenic conditions (Figure 1C,D), we questioned whether the proliferation of naïve and activated CD8^+^ T cells by IL7-Fc would be altered in non-lymphopenic and lymphopenic conditions. Therefore, naïve pmel-1 Thy1.1^+^CD8^+^ T cells were adoptively transferred to WT and RAG1^−/−^ mice. Half of these mice were immunized with mouse gp100 (mgp100) peptide in IFA to induce the activation of transferred pmel-1 CD8^+^ T cells and further treated with hIgG or IL7-Fc (Figure 5A,C). Transferred pmel-1 CD8^+^ T cells did not proliferate in non-lymphopenic WT mice who were not immunized with the mgp100 peptide; however, IL7-Fc injection-induced their proliferation in WT mice even without Ag stimulation (Figure 5A). In immune-deficient RAG1^−/−^ mice, transferred pmel-1 CD8^+^ T cells slowly proliferated in the absence of the mgp100 peptide, which was further enhanced by IL7-Fc injection (Figure 5A). Immunization with the mgp100 peptide-induced >7 divisions of pmel-1 CD8^+^ T cells in WT mice; however, this was impeded by the injection of IL7-Fc (Figure 5C). In RAG1^−/−^ mice, pmel-1 CD8^+^ T cells were proliferated by the mgp100 peptide and further by the addition of IL7-Fc (Figure 5C).

In the absence of mgp100 peptide immunization, total lymph node cells were significantly increased by IL7-Fc in WT and RAG1^−/−^ mice (Figure 5B). The percentages of pmel-1 Thy1.1^+^CD8^+^ T cells were significantly decreased by IL7-Fc in WT mice and tended to be increased in RAG1^−/−^ mice (Figure 5B). Consequently, the number of pmel-1 CD8^+^ T cells was comparable between hIgG- and IL7-Fc-treated WT mice, but significantly increased by IL7-Fc in RAG^−/−^ mice (Figure 5B). The proliferation index indicated that IL7-Fc significantly enhanced the proliferation of naïve pmel-1 CD8^+^ T cells in both non-lymphopenic and lymphopenic conditions, and was more efficient under lymphopenic conditions (Figure 5B). However, in mice immunized with the mgp100 peptide, total lymph node cells were significantly increased by IL7-Fc in WT mice, and minimally increased in RAG1^−/−^ mice (Figure 5D). In non-immunized mice (Figure 5A,B), the percentages of pmel-1 Thy1.1^+^CD8^+^ T cells were significantly decreased by IL7-Fc in WT mice and tended to increase in RAG1^−/−^ mice (Figure 5B). Nevertheless, the number of pmel-1 CD8^+^ T cells was comparable in both hIgG- and IL7-Fc-treated WT mice, and significantly increased by IL7-Fc in RAG1^−/−^ mice (Figure 5B). In mgp100 peptide-immunized mice (Figure 5C,D), IL7-Fc decreased the proliferation index of activated pmel-1 CD8^+^ T cells in WT mice, but not in RAG1^−/−^ mice (Figure 5D).

IL7-Fc significantly increased endogenous CD8^+^ T cells and the proliferation of pmel-1 CD8^+^ T cells in non-immunized WT mice (Figure 5A) and impeded the division of activated pmel-1 CD8^+^ T cells in mgp100-immunized WT mice (Figure 5B). As IL-7R expression is reduced in T cells that receive the IL-7 signal to avoid competition with unsignaled T cells for the remaining IL-7 [20], excessive amounts of IL7-Fc provoked a massive expansion of naïve and memory CD8^+^ T cells more efficiently in un-immunized mice (Figure 5A) and may have impeded the division of activated pmel-1 CD8^+^ T cells as the expanded T cells limit ‘space’ for their growth.

## 4. Discussion

As the memory potential of CD8^+^ T cells is crucial in enhancing the efficacy of ACT using T cells, we sought to determine whether the replacement of IL-2 with IL7-Fc or their combination would improve the outcome of ACT using CD8^+^ T cells. IL7-Fc significantly enhanced the efficacy of ACT using pmel-1 CD8^+^ T cells in our experimental B16-F10 melanoma model (Figure 1). IL7-Fc, particularly in combination with rhIL-2, successfully suppressed the growth of B16-F10 melanoma in mice that received activated pmel-1 CD8^+^ T cells with prior lymphodepletion (Figure 1B). However, unexpectedly, IL7-Fc accelerated the growth of B16-F10 melanoma in mice that received activated pmel-1 CD8^+^ T cells without lymphodepletion (Figure 1B). Nevertheless, IL7-Fc treatment of immune-intact mice markedly expanded endogenous CD8^+^ T cells in TLDNs and significantly increased the tumor-infiltration of endogenous CD8^+^ T cells (Figure 1C).

IL-7R (CD127) is primarily expressed in naïve and memory T cells and is downregulated in activated T cells [13,14]. Due to the limited amount of IL-7 availability in vivo [20], naïve and memory T cells that received the IL-7 signal had reduced IL-7R expression on their surface by internalizing the IL-7/IL-7R complex to avoid competition with unsignaled T cells [24,25]. Therefore, when IL7-Fc was sufficiently administered to tumor-bearing mice, there was a remarkable increase in endogenous T cells in the non-lymphopenic condition, which promoted the repopulation of endogenous T cells in the lymphopenic condition (Figure 1C,D), despite its effects on tumor growth being the complete opposite (Figure 1B).

Schietinger et al. suggested that tolerant self-antigen-specific CD8^+^ T cells fail to proliferate in response to antigens, but are temporarily rescued to proliferate under lymphopenic conditions [26]. In addition, as ~20% of human mature circulating B cells are self-reactive [27], Yu et al. proposed that the frequency of CD8^+^ T cells reacting with endogenous peptides was roughly equivalent to that of naïve CD8^+^ T cells recognizing foreign epitopes [28]. Therefore, prior lymphodepletion with CTX in our ACT model may create a favorable environment for the proliferation of self-reactive CD8^+^ T cells by inducing lymphopenia and increasing the release of tumor-associated antigens. Consequently, self-tumor Ag-specific CD8^+^ T cells have a higher chance of being activated and proliferated under lymphopenic rather than non-lymphopenic conditions, which can be further enhanced by IL7-Fc. This explains why IL7-Fc was only effective at enhancing the efficacy of ACT using CD8^+^ T cells under lymphopenic conditions.

In the cell transfer experiment, many pmel-1 CD8^+^ T cells were successfully divided only with IL7-Fc-mediated signaling in WT, even without mgp100 peptide immunization (Figure 5A). However, when WT mice were immunized with the mgp100 peptide, the transferred pmel-1 CD8^+^ T cells were less divided following IL7-Fc treatment (Figure 5C). IL7-Fc treatment not only augmented the division of transferred pmel-1 CD8^+^ T cells in non-immunized and immunized mice but also increased endogenous CD8^+^ T cells in TDLNs (Figure 5A,B). Quorum sensing is a regulatory mechanism of changes in population density [25]. Quorum sensing also operates in CD8^+^ T cells, in which a threshold population density of CD8^+^ T cells is necessary to induce BLIMP1 and the terminal differentiation of naïve CD8^+^ T cells following activation [29,30]. Therefore, the impaired division of pmel-1 CD8^+^ T cells in immunized and IL7-Fc-treated mice may be due to the regulatory effect of quorum sensing, which is turned on by the increased CD8^+^ T cell population in TDLNs.

Collectively, our data suggest that IL7-Fc preferentially enhances the antitumor effect of CD8^+^ T cells only under lymphopenic conditions, which might be due to the temporal rescue of tolerant self-tumor Ag-specific CD8^+^ T cells from peripheral tolerance. Moreover, under non-lymphopenic conditions, IL7-Fc impaired the division of activated CD8^+^ T cells due to quorum sensing in the CD8^+^ T cell population. Therefore, we conclude that IL7-Fc should be temporally administered to cancer patients under lymphopenic conditions to avoid the excessive expansion of CD8^+^ T cells that do not target tumor antigens.

## Data Availability

All data supporting the findings of this study are available within the article.
